# Improvements in Aggregate-Paste Interface by the Hydration of Steelmaking Waste in Concretes and Mortars

**DOI:** 10.3390/ma12071147

**Published:** 2019-04-09

**Authors:** Isabel Miñano, Francisco J. Benito, Manuel Valcuende, Carlos Rodríguez, Carlos J. Parra

**Affiliations:** 1 Department of Architecture and Building Technologies, Technical/Polytechnic University of Cartagena, Paseo Alfonso XIII, 30203 Cartagena, Spain; franciscojavierbenito@hotmail.es (F.J.B.); carlos.parra@upct.es (C.J.P.); 2 Department of Architecture Constructions, Polythenic University of Valencia, Camí de Vera, 46022 Valencia, Spain; mvalcuen@csa.upv.es; 3 Department of Construction Materials, Centro Tecnológico de la Construcción, Polg. Oeste, 30820 Alcantarilla, Spain; crodriguez@ctcon-rm.com

**Keywords:** self-compacting concrete, granulated slag, microstructure, mechanical properties

## Abstract

The objective of the experimental work is to study the mechanical properties in self-compacting concretes (SCC) in which part of the limestone aggregate has been replaced by granulated blast furnace slag (GBFS) in different percentages ranging from 0% to 60%. The results show that at early ages the SCC with the largest content in slag tend to have lower compressive strengths due to the poor compacting of the aggregates, although in the long-term their strength increases due to the reactivity of the slag. In fact, at the age of 365 days, the mortars made with the substitution of 50% of cement by ground GBFS reach compressive strength similar to that of the mortar made with 100% of cement. The consumption of calcium hydroxide during the hydration of the GBFS and the formation of hydrated calcium silicate (CSH) improve the mechanical properties of the slag-paste interface. The new compounds formed by the hydration of anhydrous oxides of the GBFS improve the aggregate-paste transition zone. The chemical interaction between the dissolution of the cement pore and the GBFS ends up generating new compounds on its surface. The increasing hydration of the GBFS produces a greater amount of silica gel that polymerises, densifying the matrix and reducing the porosity, which improves the mechanical properties of the concrete and perhaps its durability. The topography of the particles and their interface are analysed with atomic force microscopy techniques to assess the morphology depending on the aggregate used. On the other hand, a study was carried out of the aggregate-paste interface with scanning electronic microscope at different ages. It can be seen that in the contours of the hydrated GBFS particles, a band or ring forms with the new reaction products. The results obtained strengthen the previous conclusions. The new hydrated compounds fill the reaction ring, introducing chemical bonds between the aggregate and the interface, occupying part of the original pores or substituting spaces occupied originally by large portlandite crystals, of lesser mechanical strength and easily leached. For all this, the benefit is twofold. On the one hand, use is made of industrial by-products and, on the other hand, part of the destruction of natural quarries to obtain the necessary raw materials is avoided.

## 1. Introduction

The cement industry generates approximately 5% of the world’s CO_2_ emissions. Estimations on the use of concrete, quantify it to be as much as 30 million tonnes per year, being considered as the largest production of manufactured products [1]. Substituting natural aggregates by recycled materials or residues allows reducing CO_2eq_ (carbon dioxide equivalent) emissions (carbon footprint) in the fabrication of concretes [2,3]. This use of recycled materials or residues as aggregate in concretes allows reducing such residues accumulating in dumps [4].

The GBFS has the same chemical constituents as Portland cement, but in different proportions. More than two-thirds of the mass of the GBFS is vitreous; the sum of CaO, MgO and SiO_2_ occupies two thirds of its mass. The remainder contains aluminium oxide (Al_2_O_3_) together with small amounts of other compounds. With its abrupt cooling, upon leaving the furnace, an amorphous and chemically reactive structure is obtained, as the degree of polymerisation of the tetrahedrons (SiO_2_) is diminished, by the combination of the Al and Mg that form the vitreous phase [4]. A high percentage of Al_2_O_3_ can stimulate the development of the initial strength. The alumina or magnesium, in compounds such as melilite, once hydrated, migrate to fill the pores nearest to the interface. 

The reactive character of the GBFS can lead to a much denser microstructure [5,6], since the similar reactions to the pozzolanics contribute to reducing the content in portlandite in the matrix of the cement and increasing the content in calcium silicate hydrate (CSH). These reactions also lead to changes in the capillary network, in the porosity of the concrete, and in the interfacial transition zone [7]. These changes can lead to modifying the behaviour of the mechanical properties and durability of the SCC. 

In research by Saito et al. [8] with slag used as fine aggregate, they recorded a lower compressive strength at seven days, but point out that the results tend to equal out at both 28 and 91 days due to the latent hydraulicity of the slag. Conversely, in the trials carried out by Demirboğa and Gül [9] with basic nature slag and good hydraulic activity, it is observed that substituting 70% of the coarse aggregate gives rise to improvements in compressive strength of up to 87% for different ratios of aggregate/cement, even at seven days of age. These concretes also present a greater tensile strength, less absorption, and a higher elasticity modulus. Similarly, Krishnasami and Malathy [10], Scandiuzzi and Battagin [11] and Valcuende et al. [12] obtained greater strengths in the long term due to the hydraulic activity of the slag.

The objective posed in this experimental work is to study the microstructure and compressive strength of SCC with GBFS as fine aggregate and with cements with low clinker content. With this, a use is given to an industrial by-product, whose storage and treatment creates serious problems in the environment and at a high cost. Moreover, the amount of natural aggregate used in the dose is reduced, with corresponding saving in natural resources, contaminant emissions into the atmosphere and energy necessary for its extraction. Additionally, the production of SCC with cement CEM II/B-M (S-L) 42.5R, with a quantity of clinker of 274 kg/m^3^ and the application of secondary filling materials such as ground slag and fine limestone (115 kg/m^3^), contributes to further reduction in the emission of CO_2_ into the atmosphere.

## 2. Experimental Programme

### Materials

Seven types of self-compacting concretes were produced: a reference one (H-0) and six with different substitutions of the fine aggregate by blast furnace slag (10%, 20%, 30%, 40%, 50% and 60%), all of which were fabricated with a ratio aggregate/cement of 0.55. The characteristics of each mix are shown in Table 1. 

**Figure 1:**
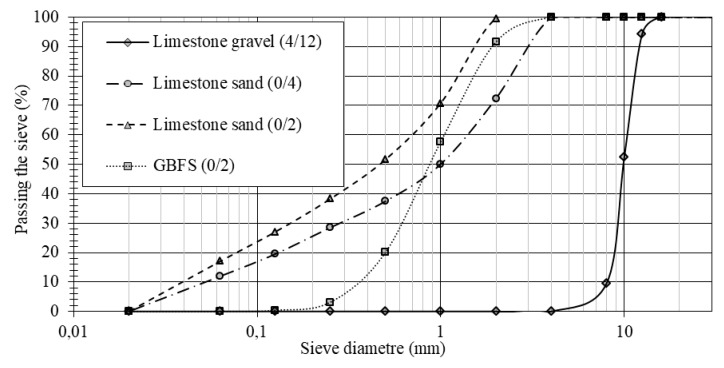
Granulometric curves of the different aggregates.

All the mixtures studied were made with 375 kg/m^3^ of cement CEM II/B-M (S-L) 42.5R and 25 kg/m^3^ of fly ash. The granular skeleton is constituted by three fractions of crushed limestone aggregate: gravel 4/12 mm, coarse sand 0/4 mm and fine sand 0/2 mm, with a content of fine (particles < 0.063 mm) of 1.0%, 12.0% and 17.1%, respectively (Figure 1). In the concretes with slag, part of the fine sand was substituted by slag with a granulometric fraction of 0/2 mm. Limestone filler was added to these concretes to compensate the lack of fine sands in the slag.

The granulated blast furnace slag used has a high content in SiO_2_ and CaO, of basic nature. The additive used was a base of polycarboxylate superplasticiser (Viscocrete 3425).

**Figure 2:**
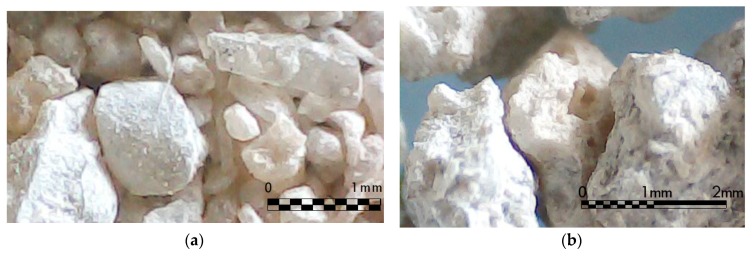
Micrographs of samples of granulated blast furnace slag.

The GBFS is formed by small alveolar particles with cutting edges. The grains of slag have an angular form and present medium sphericity. In the micrograph of Figure 2, the translucent state by the effects of vitrification of the small particles and/or fragments of GBFS can be observed. The main chemical composition of GBFS is SiO_2_ (34.41%), CaO (41.9%) and MgO (7.18%). 

## 3. Tests and Methodology

### 3.1. Fresh State Tests

**Figure 3:**
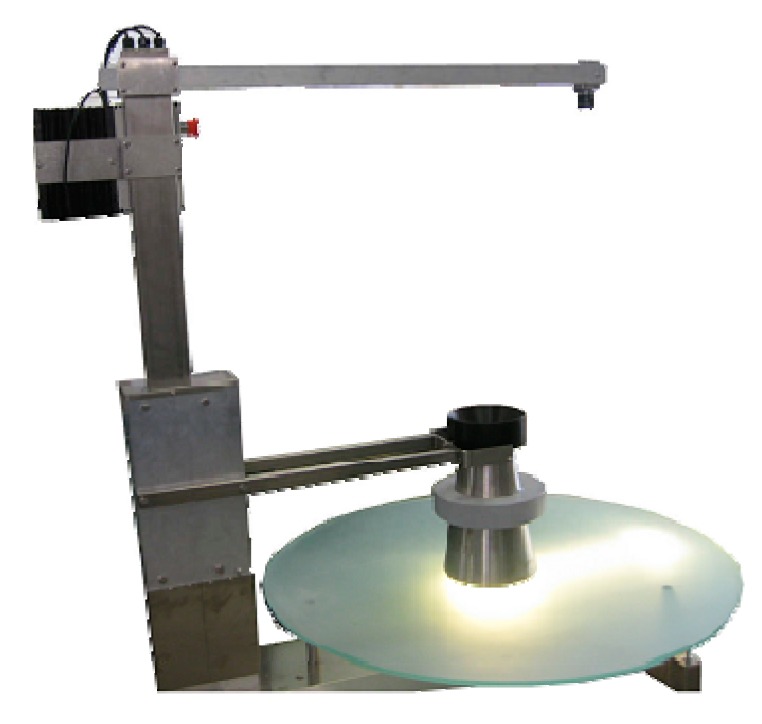
4C-Rheometer.

In the experimental phase, the rheometric device 4C-Rheometer was used (Figure 3), to determine the fluidity and characteristics of the fluid using two parameters: plastic viscosity and shear stress, both of which are used in Bingham’s theoretical models. The results of the 21 batches analysed are shown in Table 2. All the self-compacting concretes with substitution of fine aggregates by granulated blast furnace slags present good workability, with results in the drainage test of Øf equal to 700 ± 30 mm and with T_500_ quite low, and good resistance to segregation.

### 3.2. Tests of Compressive Strength of Mortars and SCC with Slag

In order to study the hydraulic activity of the slag, four mortars were fabricated with reference dose 1:3:0.5 (Type I Portland cement: sand: water): one of reference without slag (M-1), another in which half of the cement was substituted by ground slag with granulometry similar to that of the filler (M-2), another in which half of the cement was substituted by limestone filler (M-3), and finally another with only half the cement without the addition of limestone filler nor slag (M-4). The cube tests were assayed at the ages of 2, 7, 28, 90 and 365 days. Three batches were made for each mix. For each batch, two samples were carried out, with the result of each test being the arithmetic mean of the six values obtained.

Likewise, two cylindrical test were prepared with 150 mm diameter and 300 mm high of each batch for the test of compressive strength at 7, 28, 90 and 365 days (EN 12390-3:2003 [13]).

## 4. Result and Discussion

### 4.1. Reactivity of the Finely Ground Slag

#### 4.1.1. Chemical Composition of the Slag

**Figure 4:**
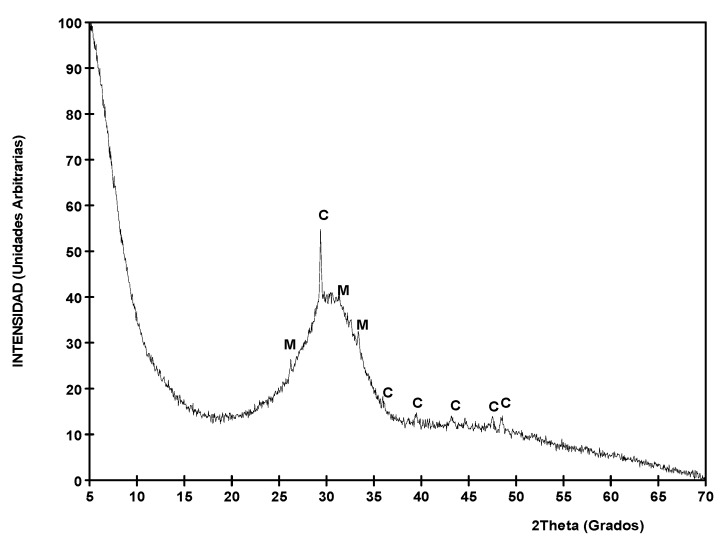
Diffractogram of X rays of the GBFS.

The slags used in this work are considered potentially hydraulic according the CaO/SiO_2_ basicity index equal to 1.22 [14]. This reactivity of the slag is also appreciated in the X ray diffraction assay (Figure 4) since the diffractogram obtained is characteristic of an amorphous slag, where there are almost no peaks of crystalline minerals, but there is a hump around 30 degrees (2Theta). This angle corresponds to the principal peak of melilite, which is the principal mineral of the crystallised slag. The small peaks that are detected are of calcite (CaCO_3_) and mullite (3Al_2_O_3_·2SiO_2_).

#### 4.1.2. Compressive Strength of the Mortars with Slag

**Figure 5:**
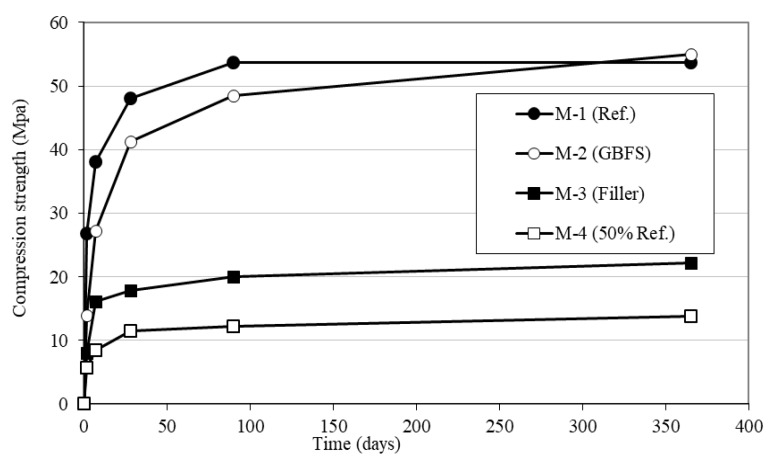
Compressive strength of the mortars (2, 7, 28, 90 and 365 days).

**Figure 6:**
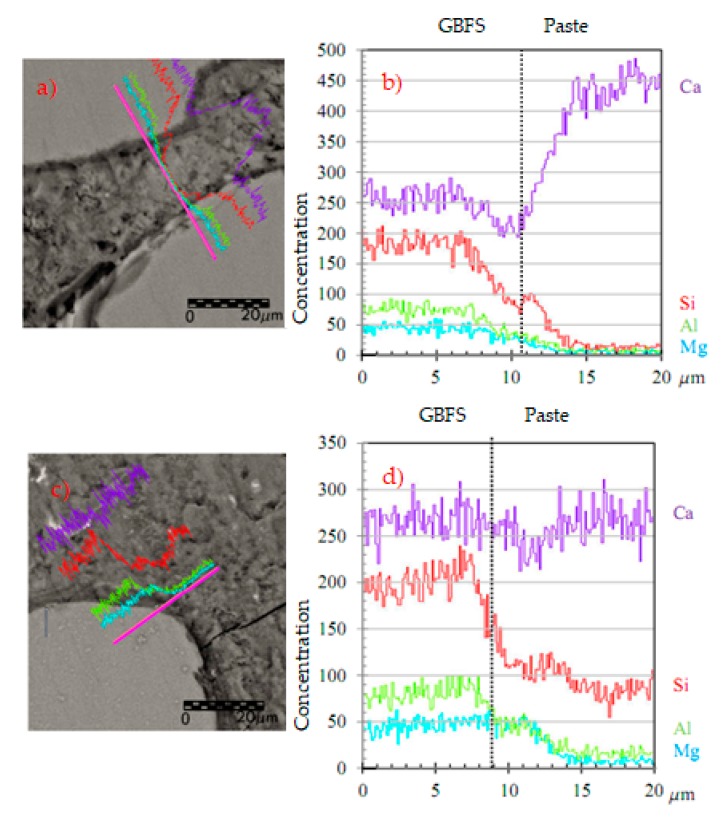
(**a**) SEM images of the interface at 1300 magnification to 28 days; (**b**) the analysis of the composition to 28 days; (**c**) SEM images to 120 days; (**d**) the analysis of the composition to 120 days.

In Figure 5, it is observed that the mortar fabricated with 50% less cement (M-4) than the standard mortar (M-1) presents, as expected, a major loss in important strength, in the order of 77%. However, if the part of the cement eliminated is substituted by fine limestone (M-3), the loss in strength at young ages is not so important, since the fine occupies the spaces available and makes the distribution of particle broader, providing a denser cementing matrix (filler effect). In the hydrated cements, small amounts of additions of limestone increase the volume of the solids [15] and a small part of the CaCO_3_ of the filler promotes the reconversion of monosulphoaluminate in ettringite, provoking an increase in the total volume of the hydrated phase [12,16]. In the interfacial transition zone of the slag mixtures have been found (in the SEM images) some hydrated compounds that have not been found in the rest of the paste, such as for example Mg (Figure 6). The Mg, coming from the hydration of the slag, diminishes its concentration as the distance to the GBFS increases and tends to disappear to a few microns. On the other hand, at early ages, it can be seen that the loss of strength is somewhat less since the fine limestone accelerates the hydration of the cement as it provides the additional surface area to ease the nucleation and creation of hydration products [17,18]. This effect is reduced at more advanced ages. Thus, for example, at the age of two days, the loss in strength in relation with the standard mortar is of 52% and yet at the ages of 28, 90 and 365 days, the loss in strength remains practically constant, in the order of 62%.

It can also be appreciated from Figure 5 that the strength of the mortar with slag (M-2) is far superior to that of the mortar with fine limestone, with the difference between them being increasingly greater as the age of the cube test increases. In other words, the finely ground slag is clearly reactive. However, the hydration of the slag is rather slow, since in the first days a large part slag remains inert, with the strength of the mortar at the age of two days being in the order or 50% of the strength of the standard mortar (mortar with 100% of cement, M-1). Nevertheless, with the pass of time, the hydration reactions progress and calcium silicate hydrates are formed, reaching the strengths of the standard mortar after one year. 

#### 4.1.3. Study of the Aggregate-Paste Interface with Scanning Electronic Microscope

On the other hand, a study was carried out of the aggregate-paste interface with scanning electronic microscope at different ages with the objective of seeing the evolution in the reactivity of the slag and its participation in the strength phase of the microstructure. Figure 6 shows the GBFS-paste interface at 28 and 120 days (magnification ×1300) and its comparative analysis of the composition of significant elements (Ca, Mg, Al and Si). In this analysis and in the representation of the mapping in Figure 6, the presence of Magnesium in the paste can be seen together with the GBFS (interface). 

The band of the highest concentration of Al and Mg atoms that have migrated from the GBFS to the interface is around 3 µm wide at 28 days and 5 µm at 120 days (Figure 6). The presence of Al and Mg in the slag-paste interface is very significant to determine the reactivity of the GBFS due to the almost null presence of these elements in the original composition of the paste.

**Figure 7:**
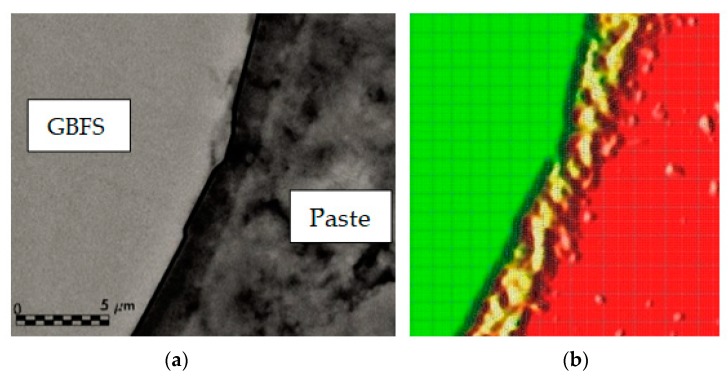
(**a**) Interface at 28 days; (**b**) representation of the mapping of Mg (band of 3 µm in yellow).

In the SEM images, it can be seen that in the contours of the hydrated GBFS particles a band or ring forms with the new reaction products (Figure 6 and Figure 7). In this zone, greater concentrations of Al, Mg and Si are obtained than in the matrix and somewhat less than in the GBFS particle itself, which is what provides the different oxides of Al, Mg and Si. The oxides upon dissolving with the alkali of the cement react forming new gels of CSH, some of the tobermorite type or with a structure similar to it, as well as other structures of lesser Si/Ca proportion. The tobermorite gel is responsible for the resistant microstructure of the cement paste, as well as for the adherence it has with the aggregates [19]. 

**Figure 8:**
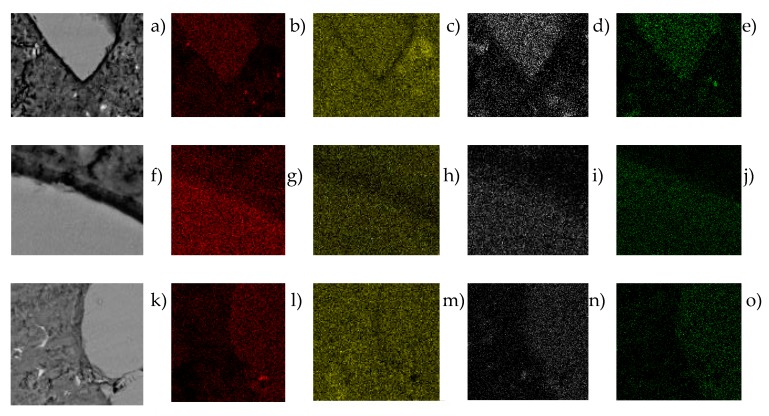
(**a**) SEM image at 28 days at 1300×; maps of electrons of Si (**b**), Ca (**c**), Al (**d**) and Mg (**e**); (**f**) SEM image at 28 days at 5000×; maps of electrons of Si (**g**), Ca (**h**), Al (**i**) and Mg (**j**); (**k**) SEM image at 120 days at 1300×; maps of electrons of Si (**l**), Ca (**m**), Al (**n**) and Mg (**o**).

The alkalis of the paste attack the surface of the GBFS particles and allow polymerisation reactions to develop while anhydrous GBFS dissolution exists. Figure 8 shows, at 1300 and 5000 magnifications, different slag particles that had not totally dissolved at 28 and 120 days. In all of them, the reaction bands or rings can be observed around the particle, in a clearly delimited zone, generally of a darker tone in the SEM images obtained. Moreover, in the mapping, it is checked how in this zone of rings the amount of Ca diminishes due to the greater porosity of this zone of the interface. Additionally, in this zone, the concentration of Al and Mg suffers a significant spike with respect to the matrix, coming from the first compounds that have been formed in the interface by the hydration of the slag. The hydration ring is clearly detected in Figure 7a in a band of 3 µm next to the slag, similar to that represented in Figure 7b, where the maximum concentrations of Mg are represented in the reaction ring, in a 3 µm wide band.

At 28 days, the concentration of Al in the GBFS particles is much greater than in the reaction ring around it, due to the fact that the slag has still not totally dissolved and can continue generating new silicate hydrates in adequate activation conditions. In other words, with the generation of new crystals of Ca(OH)_2_ with the passing of time and high pH, the continuity of the dissolution of the anhydrous compounds of the GBFS (principally oxides of Si, Ca, Al and Mg, Figure 8) is favoured, which enables the formation of more CSH. This is thanks to the amorphous slag composition used, where there are almost no peaks of crystalline minerals (Figure 4), which favors the hydration of the slag and the continuity of the dissolution of the anhydrous compounds of the GBFS. At 120 days, the dissolution of the anhydrous slag remains active and the reaction ring is extended to more than 5µm. At 120 days, the contours of the aggregates are less defined than at 28 days. This is due to the dissolution of the anhydrous oxides in the contours of the slag, which hydrate and migrate to the reaction ring. That is to say, the GBFS borders are blurred by the hydration, improving the mechanical properties of the interface zone by introducing chemical bonds between the slag and the paste. The chemical interaction between the dissolution of the pore of cement and the GBFS ends up generating new particles on its surface. The paste is richer in Si the closer it is to the surface of GBFS particles, which together with the chemical change produced on the surfaces of these aggregates means that the interface behaves like a strong bond, which leads to better mechanical properties.

The mapping of Al and Mg for all the samples analysed at 28 and 120 days indicates that a large amount of the slag did not dissolve, with anhydrous material, oxides without reaction such as periclase (MgO) or alumina (Al_2_O_3_) remaining. On the other hand, it is observed that Al, Mg and Si are more abundant in the GBFS particle than in the ring and in the matrix, highlighting a spot increase in the ring zone for Al and Mg due to the greater proximity to the zone where the anhydrous compounds from the slag are dissolved (Figure 8). Finally, the presence of Ca is shown in similar amounts between the slag particle and in the matrix (contributed by the Portland cement), with reductions in Ca in the ring zone due to its greater porosity, which translates into less calcium silicate hydrates, similar to that described for the reduction in Si in the zone nearest to the GBFS. 

The mappings of Si and Ca in the samples at 120 days show a smaller difference between the concentration in the particle and in the matrix, due to the greater hydration of the slag with the passing of time. On the other hand, the mapping of Al shows a greater concentration in the particles, probably due to the presence of mullite without reaction.

#### 4.1.4. Elemental Proportions of Al/Si and Si/Ca

**Figure 9:**
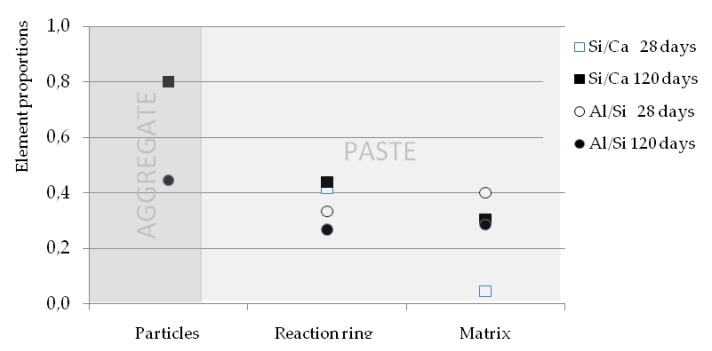
Element proportions of Al/Si and Si/Ca in the interface at 28 days and 120 days.

On the other hand, in the samples analysed, the elemental proportions of Al/Si and Si/Ca were obtained (Figure 9). The Si/Ca proportion in the matrix and in the ring of the samples analysed with GBFS could indicate that, in part, CSH is being formed with a structure similar to that of tobermorite, or even that tobermorite and other structures of lower Si/Ca proportion are being formed. However, the Al/Si proportion is lower in the reaction ring, possibly due to the Al reacting faster than the Si in the particles and is incorporated into the CSH of the matrix, as well as the slightly higher content of Al in the matrix than in the ring [19]. The Al/Si proportion of the particles of slag is similar to that of the matrix. The slight descent in the Al/Si content of the matrix at 120 days with respect to 28 days is brought about principally by the greater amount of Si in the matrix of this sample, by the CSH generated. The difference of the Al/Si proportion of the particles with that of the reaction ring is due to the presence of phases without reacting, that is to say anhydrous material with the possibility to continue generating new silicate hydrates. The difference of concentration at 28 days of the Al and Si is of 260% and 133%, respectively. At 120 days, the difference is of 200% and 120%, respectively. The reduction in the differences of concentration with the passing of time between the particle and the reaction ring is produced by the continuation of the hydration of the slag, as was confirmed in other trials, such as in the mortars tested at compressive. The relations with silicate aggregates shown in Figure 9 are not comparable with those of the GBFS, as they do not have a different composition, although it provides a reference of the matrix and the reaction ring without hydrated compounds of the GBFS. The non-presence of Ca in the particles of silicate aggregate makes the Si/Ca proportion rise. In the reaction ring, it is 0.61, descending to 0.39 in the matrix.

At 28 days, the Si/Ca proportion of the GBFS particle is 0.80 (Figure 9), whilst in the interface zone nearest to the above, in the reaction ring, the relation falls to 0.42, reaching 0.04 in its minimum point of the matrix; as the amount of Ca rises rapidly due to, among others, the Ca(OH)_2_ present in the zone, as well as substantially diminishing the concentration of Si (from 100 to 20 units) for the above-mentioned conjunction of two adjacent interfaces, increasing the porous zone and lowering the amount of silicates that are formed in proportion with crystals of portlandite that are generated. At 120 days, the Si/Ca proportion of the reaction ring (0.44) increases slightly with respect to that at 28 days. The greatest differences between both ages are seen in the matrix, passing from 0.04 to 0.30, largely due to the greater reach of the diffusion of the new silicates generated by the slag, while the consumption of Ca(OH)_2_ increases. In the X ray diffraction tests on the GBFS particles, it was obtained that 34.41% is composed of silicate oxides (SiO_2_) and 41.9% of Calcium oxides (CaO), which gives a SiO_2_/CaO proportion of 0.82, identical to the Si/Ca proportion obtained in the analysis with SEM-EDS in the zone of the GBFS particle.

Another point of interest is the difference that exists between the Al/Si proportion of the matrix and the reaction ring. The zone furthest from the matrix has a slightly higher content of Al than in the part of the ring furthest from the GBFS (Figure 9), since the Al reacts before the Si of the particles and is incorporated into the CSH of the matrix. This causes the surfaces of the particles that are reacting to have a higher content of Si and thus the Al/Si proportion is lower in the ring.

The investigation of Wang et al. [20] establish that the relation a decrease of the ratio Si Al of the slag can increase the reactivity of the slag cooled to the air and its degree of reaction, due to a greater release of heat of hydration, a lower content of CSH, a greater amount of combined chemical water and a denser microstructure.

### 4.2. Analysis of Topography of the Interface with Atomic Force Microscopy 

Inspections are carried out with atomic force microscopy techniques on the samples previously analysed using SEM. The forces of interaction that exist between cantilever (inspection lever) and the sample allow the topography of the sample to be obtained. Moreover, images of the phase are obtained that enable to detect, generally in a qualitative manner, variations in the chemical compositions, adhesion, or other surface properties of the sample that are not necessarily manifested in topographic images. The topography of the particles and their interface are analysed to assess the morphology depending on the aggregate used. The contour of the aggregates is perfectly delimited in the samples. 

**Figure 10:**
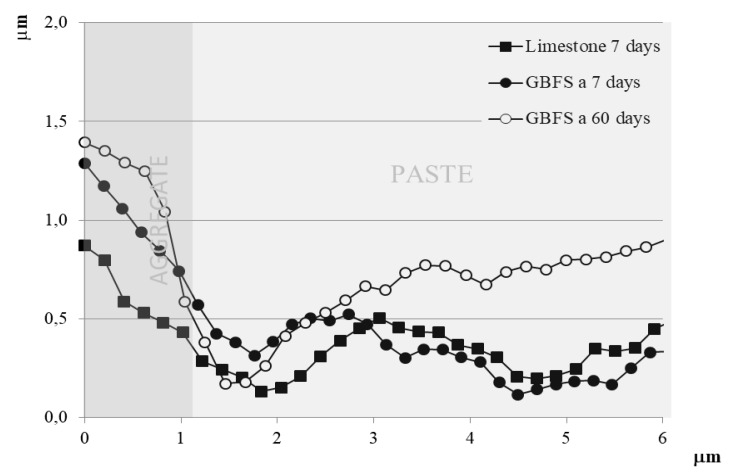
Topographical profile of the interface at 7 and 60 days with atomic force technique.

The results obtained strengthen the previous conclusions. The matrix zone is more sunken than the aggregates by around 0.5 µm. The junction zone between the aggregate and the matrix is at a still lower height than the paste. This lower height zone of the aggregate-paste interface is smaller in size in the GBFS sample at 60 days than at 7 days. No major differences can be appreciated between the sample with limestone aggregate and that with GBFS at 7 days (Figure 10). However, at 60 days, when most of the improvements in strength of the mortars and concretes fabricated have occurred (Section 4.1.2), the topographical profile undergoes a significant increase in height in a band of around 5 µm wide. This band is very similar to that detected with a greater concentration of atoms of Al and Mg at 120 days, which could verify the filler of the porous network in that zone, densifying the zone and increasing the compressive strength, as seen in the trials of Section 4.1.2. In the acquisition of greater strengths of the particles, the formation of new solid crystals intervenes from the anhydrous oxides of Si, Al and Mg of the GBFS. The new hydrated compounds, as has been shown in the rest of the tests, fill the reaction ring, introducing chemical bonds between the aggregate and the interface, occupying part of the original pores or substituting spaces occupied originally by large portlandite crystals, of lesser mechanical strength and easily leached. All this improves the mechanical properties of the zone, affecting the detachment of particles by abrasion of the polishing. 

**Figure 11:**
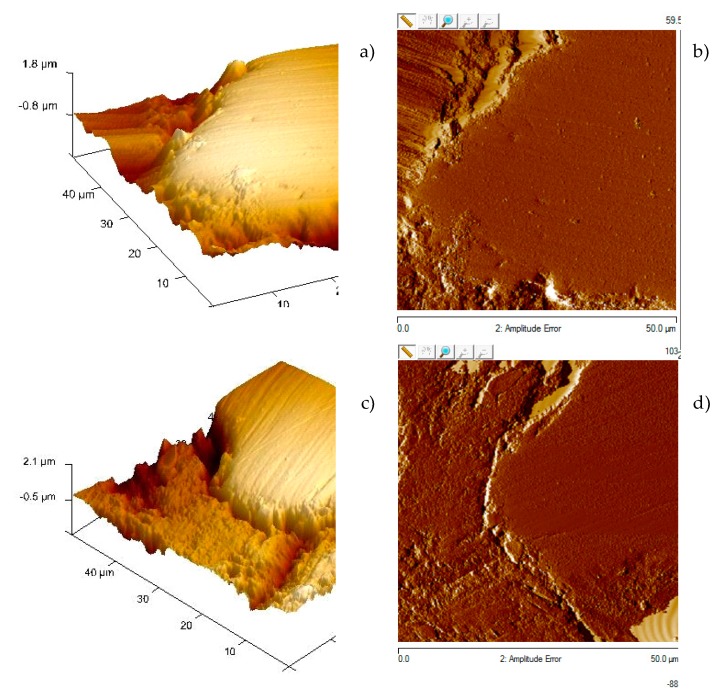
(**a**) Graphical representation of the topography sample with limestone aggregate, (**b**) changes of its phase in the interface, (**c**) graphical representation of the topography sample with GBFS, (**d**) changes of its phase in the interface.

A representative graphic of the topography of two of the inspections carried out is shown in Figure 11. In both samples, the inspected aggregates are perfectly delimited, with the paste around 0.5μm more sunken than the aggregates after the polishing of the sample. The join zone between the aggregate and the paste (interface), due to its lower resistance and the greater detachment of particles during polishing ends up at a lower height than the paste. This lower zone of the aggregate-paste interface is slightly wider in the sample of limestone aggregate (above) than in the GBFS (below). Moreover, this same conclusion is reached by analysing the information regarding changes of phase, which allows variations in the chemical composition, adhesion or other properties of the sample’s surface to be qualitatively detected, with a lower width in the interface in the sample with GBFS being detected (Figure 11a,c). 

### 4.3. Compressive Strength of the SCC

**Figure 12:**
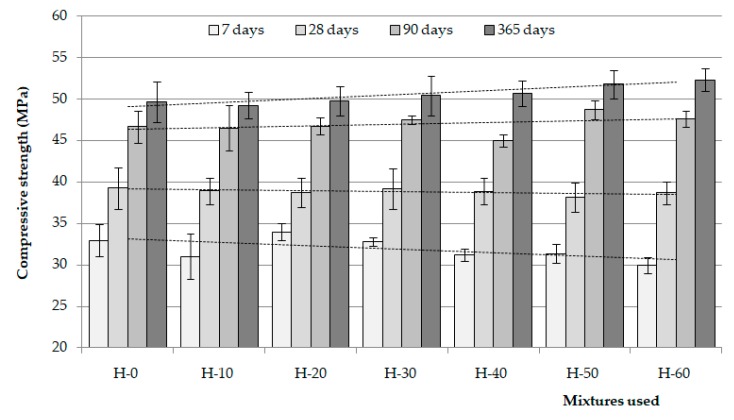
Histograms with dispersions of results.

In the first seven days, the results of compressive strength are very similar, although the concretes with higher contents of slag tend to give slightly lower strengths (Figure 12). Bearing in mind that the content of cemented paste is the same in all the doses, this may be due to poorer compacting of the aggregates, since the slags have a very angular shape and present a granulometry with scant presence of particles smaller than 0.5 mm, which may give rise to mixes with less compactability. 

At more advanced ages, the behaviour is different. The slags used are reactive and creates CSH around the particles with hydration. This densifies the interface, improves aggregate-paste adherence and reduces the negative effects produced from worse compacting of the slags. The mixes with more substitution of sand by slag present, in general, a finer porous structure probably due to the greater formation of CSH. However, these hydration reactions are very slow (Section 4.1.2) and, therefore, the improvement in the mechanical behaviour is also slow. At ages of 28 and 90 days, the compressive strength of all the concretes is similar, with no statistically significant differences existing (*p*-value = 0.73 at 28 days and *p*-value = 0.19 at 90 days, for a confidence level of 95%). On the contrary, at the age of 365 days, it is observed that the higher the content of slag used as aggregate, the higher the strength of the concrete (*p*-value = 0.04). 

## 5. Conclusions

From the experimental work carried out, the following conclusions can be established:

(1) Participation of the GBFS. The formation of hydrated products (CSH) from the paste is confirmed. At the age of 365 days, mortar with substitution of 50% of cement by ground GBFS reaches a compressive strength, similar to that of the reference mortar, with 100% cement.
(2) New compounds formed. At 120 days, the greatest concentration of atoms of Al and Mg is obtained in the slag-paste interface, in a band of around 5 µm around the GBFS. The new compounds improve the aggregate-paste transition zone, introducing chemical bonds between the GBFS and the paste. The chemical interaction between the dissolution of the pore of cement and the granulated blast furnace slag ends up causing new particles on the surfaces. The paste is richer in Si the closer it is to the surface of the aggregate particles (GBFS), which together with the chemical alteration that occurs on the surfaces of these aggregates means that finally the interphase behaves like a strong bond, which implies an improvement in mechanical properties.
(3) Improvements of the slag-paste interface. The mechanical properties are improved by the hydration of the slag. Calcium hydroxide is consumed, of lower adherence, and more strength than the CSH, and more CSH is formed.
(4) The compressive strength. At 7 days, the SCC with the greatest content of slags tends to have lower strength due to a poorer compacting of the aggregates. However, at 365 days, due to the reactivity of the slags, the higher the amount of sand substituted by slag, the greater the compressive strength of the concrete tends to be and a greater relative increase of the compressive strength at 28 and 90 days, with respect to the initial strength at 7 days.
(5) As a general conclusion, all of the above allows to fabricate SCC with a reduction in energy consumption and raw materials, as well as a decrease in greenhouse gas emissions. The benefit is two-fold. On the one hand, use is made of industrial by-products and, on the other hand, part of the destruction of natural quarries to obtain the required raw materials is avoided. The SCC with GBFS as fine aggregate are viable from the point of view of sustainability and possibly valid for structural use.

The results obtained make it interesting to continue studying the durability of these concretes for a better global analysis of the results.

## Figures and Tables

**Table 1:** Dosage of the concretes.

Mix	Limestone Sand (0/4) kg/m^3^	Limestone Sand (0/2) kg/m^3^	Gravel (4/12) kg/m^3^	GBFS Aggregate (0/2) kg/m^3^	Cementing Material		Limestone Filler kg/m^3^	Superplast kg/m^3^	Water l/m^3^
					Cement kg/m^3^	Fly Ash kg/m^3^			
H-0	861.3	274.1	656.6	0.0	375	25	0.0	4.4	220
H-10	870.3	159.0	656.8	100.0	375	25	32.5	4.2	220
H-20	873.2	44.7	656.3	199.1	375	25	64.7	4.0	220
H-30	794.6	0.0	656.3	303.1	375	25	91.5	4.6	220
H-40	686.2	0.0	656.3	410.5	375	25	114.6	4.8	220
H-50	560.1	0.0	655.7	515.2	375	25	137.0	7.2	220
H-60	456.6	0.0	663.9	619.3	375	25	159.3	6.0	220

**Table 2:** Rheological data.

Batch	H-0			H-10			H-20			H-30			H-40			H-50			H-60		
	A-1	A-2	A-3	A-4	A-5	A-6	A-7	A-8	A-9	A-10	A-11	A-12	A-13	A-14	A-15	A-16	A-17	A-18	A-19	A-20	A-21
Viscosity µ (Pa·s)	19	17	14	14	21	22	42	43	42	34	35	35	41	27	58	24	32	46	11	21	11
Shear stress τ (Pa)	15	17	22	15	15	20	20	18	20	26	16	22	20	18	26	17	24	17	17	14	14
Øf (mm)	718	700	670	720	720	680	680	690	680	660	707	670	680	691	650	700	660	703	700	723	728
T_500_ (s)	1.3	1.2	1.2	1.1	1.4	1.4	2.3	2.2	2.2	1.9	1.7	1.9	2	1.8	3.4	1.4	2.2	2.5	1.2	1.3	1
